# Evaluation of a Commercial Device Based on Reflection Spectroscopy as an Alternative to Resonance Raman Spectroscopy in Measuring Skin Carotenoid Levels: Randomized Controlled Trial

**DOI:** 10.3390/s23177654

**Published:** 2023-09-04

**Authors:** Jeong-Eun Hwang, Jin-Young Park, Myoung Hoon Jung, Kunsun Eom, Hyun Seok Moon, Hyojee Joung, Yoon Jae Kim

**Affiliations:** 1 Device Research Center, Samsung Advanced Institute of Technology (SAIT), Samsung Electronics Co., Ltd., Suwon 16678, Republic of Korea; je.hwang@samsung.com; 2 Department of Public Health Science, Graduate School of Public Health, Seoul National University, Seoul 08826, Republic of Korea; hjjoung@snu.ac.kr; 3 Health H/W R&D Group, Device eXperience (DX), Samsung Electronics Co., Ltd., Suwon 16678, Republic of Korea; jya.park@samsung.com (J.-Y.P.); mh5.jung@samsung.com (M.H.J.); k.eom@samsung.com (K.E.); hs4341.moon@samsung.com (H.S.M.)

**Keywords:** skin carotenoids, resonance Raman spectroscopy, reflection spectroscopy, diet intervention, evaluation

## Abstract

Resonance Raman spectroscopy (RRS) has been used as a reference method for measuring skin carotenoid levels (SCL), which indicate vegetable and fruit intake. However, RRS is not an easy-to-use method in SCL measurement due to its complicated implementation. In this study, a commercial spectrophotometer based on reflection spectroscopy (RS), which is relatively simple and inexpensive, was evaluated to confirm usability compared with RRS in measuring SCL. To investigate the agreement between RS and RRS, eighty participants were randomly assigned to a high-carotenoid diet group (21 mg/day of total carotenoids) or a control-carotenoid diet group (14 mg/day of total carotenoids) during a 6-week whole-diet intervention period and a 4-week tracking period. Strong correlations between the RS and RRS methods were observed at baseline (r = 0.944) and the entire period (r = 0.930). The rate of SCL increase was similar during the diet intervention; however, the initiation of the SCL decrease in RS was slower than in RRS during the tracking period. To confirm the agreement of RS and RRS from various perspectives, new visualization tools and indices were additionally applied and confirmed the similar response patterns of the two methods. The results indicate that the proposed RS method could be an alternative to RRS in SCL measurements.

## 1. Introduction

Carotenoids are important indicators of vegetable and fruit (V & F) intake because they cannot be produced in the human body and must be obtained via external intake [1]. V & Fs are a major source of carotenoids, the consumption of which has numerous health benefits, such as antioxidant effects and a lowered risk of metabolic syndrome, cardiovascular disease, cancer, and potentially all-cause mortality [2,3,4]. The ingested carotenoids are transported via the bloodstream and accumulate in various organs, particularly the skin, and prevent premature aging and malignancy [5,6]. To measure carotenoid levels in humans, blood or skin tissue is collected and analyzed [7,8]. High-performance liquid chromatography (HPLC) for blood is performed to detect serum carotenoids, which are related to the short-term dietary intake [5]. However, owing to the light-sensitive characteristics of carotenoids, blood carotenoid quantification involves experimental difficulties. Further, the standard materials and equipment are generally expensive [9,10]. Furthermore, the quantification of carotenoids in skin tissue using HPLC is difficult to conduct because a biopsy of a relatively large tissue volume is required [8].

Therefore, noninvasive methods of skin carotenoid level (SCL) measurement have been proposed to estimate V & F intake [8,11,12]. In particular, resonance Raman spectroscopy (RRS) has been used as a reference method in several studies [5,8,13,14,15,16,17,18]. RRS is an optical method used to measure the concentration of carotenoids in the stratum corneum of human skin [1]. A laser with a wavelength of 488 nm is radiated on the skin, and the spectrum of reflected light contains a carotenoid-related peak superimposed on a fluorescence background [5]. The size of the RRS peak correlates with results from the HPLC of skin biopsies [8,13,14], and the heel is recommended as an ideal site for a biopsy because of its thick (1–2 mm) and bloodless stratum corneum. Cross-sectional studies have observed a relationship between V & F intake and SCL measured using RRS [15,16]. Dynamic responses to V & F intake and stress factors such as fatigue, illness, smoking, and alcohol consumption are also investigated using RRS [17,18]. However, existing RRS is a complicated method, as it requires various optical components including a laser source and high-performance light receiver. Thus, the device structure is complicated and expensive, and it requires internal calibration. Furthermore, the detailed conditions of RRS must be set by a trained researcher, and it is difficult to apply them universally owing to different setting conditions and incompatible scales.

Another noninvasive method, a relatively simple and implantable method known as reflectance spectroscopy (RS), has been employed to measure SCL. In the 1960s, predicting carotenoids in human skin using RS with wavelengths near 480 nm was suggested [19]. Since the late 1990s, RS has been conducted on human skin, and the results exhibited a significant correlation with those of the references, HPLC and RRS [17,20,21,22,23,24,25]. Since the 2010s, standalone RS devices optimized for SCL measurement have been implemented and used for V & F intake monitoring [17,21,22,23,24,25]. Recent attempts have been made to minimize the effect of the blood or skin tone, and RS devices using numerous light sources and detectors have also been proposed [26,27]. While RS devices with a single point cannot distinguish between signals with different light paths, the light-emitting diode (LED)–photodiode (PD) structure based on the concept of spatially resolved spectroscopy accumulates photons at several positions that have different light paths [28,29,30,31]. The device consists of 118 light emitters and 152 light detectors; almost 18,000 raw data points with separated light paths could be extracted in one trial [26]. The SCL measured by the RS-based device was highly correlated with those measured by RRS (r = 0.83) [26] and the serum total carotenoid (r = 0.68) [3].

In this study, we proposed the measurement of SCL by using a commercial spectrophotometer based on RS. To obtain a carotenoid-specific signal, wavelengths of the range related to carotenoids are selected by referring to the study by Ermakov and Gellermann [21]. Additionally, the effect of blood should be considered to increase the measurement accuracy. Jilcott et al. [24] proposed a pressure-mediated method of removing blood noise; however, the method is inconvenient and not quantitative. Therefore, the problem of canceling blood volume variance is solved by adding one hemoglobin-related wavelength. The RRS method is used as a reference, as in previous noninvasive studies. This is because conducting skin biopsies every week as a reference poses significant harm to the participants and is challenging to justify from a research ethics standpoint.

To verify a commercial RS-based spectrophotometer, a carotenoid diet intervention was performed in this study. Additional approaches were considered for evaluating RS as an alternative to RRS. The degree of agreement between two different measurement approaches has been conventionally evaluated as correlations or Bland–Altman plots [11,32,33]. Although they are useful in cross-sectional studies, they are ineffective in evaluating the agreement of dynamic responses to diet interventions. Furthermore, the *t*-tests in previous studies only account for significant changes in average values but do not explain the proportion of participants who have changed in the direction intended by the intervention. To evaluate individual responses, not only statistical indices of the group but also the level of respective participants should be considered. Thus, a novel visualization tool and indices were further applied to evaluate the agreement between RS and RRS results from more diverse perspectives, including changes at an individual level.

## 2. Materials and Methods

### 2.1. Diet Intervention Study Design

**Figure A1:**
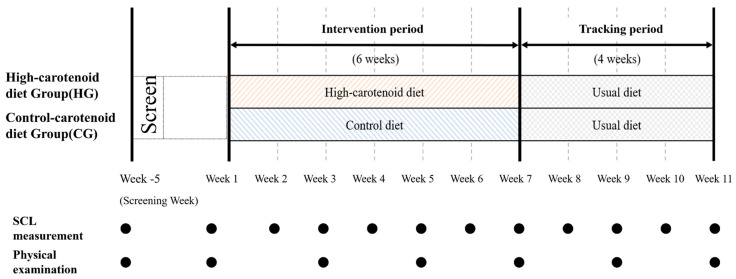
Study design and process. Skin carotenoid levels (SCL) were measured weekly during the screening week (5 weeks before the intervention), 6-week intervention periods, and 4-week tracking periods.

The study was designed as a randomized, parallel-group, whole-diet controlled trial involving a 6-week dietary intervention and a 4-week tracking period (Figure A1). This study was approved by the Seoul National University (SNU) Institutional Review Committee (No. 2010/001-014, Approval date: 12 October 2020) and was officially registered under KCT0005702 at https://cris.nih.go.kr (Registered date: 24 December 2020). Since the start of the study, there have been no significant changes in the procedure.

Korean adults aged over 18 without underlying diseases were recruited at SNU, and screening tests (at week 0) were conducted five weeks before the start of the intervention (at week 1). SCL, body mass index (BMI), and waist circumference were measured to analyze their initial status in week 0 and week 1. The participants were informed that they could withdraw from the study at any time if they were willing to stop participating, developed symptoms related to COVID-19, or came into contact with an infected individual. After screening based on eligible criteria (Supplemental Table S1), 80 participants were stratified by SCL, age, gender, and BMI and then randomly assigned to the HG or the CG at a 1:1 ratio using computer-generated random numbers. The staff who measured or analyzed the outcome variables were blinded to the allocation. All participants were blinded to their allocation groups and the intervention study before the intervention. Although participants could infer their allocation through their diets, researchers did not inform them of the allocation results until the study was completed.

During the 6-week intervention period, the HG was provided with a high-carotenoid diet (including average total carotenoids contents: 21.0 mg/2000 kcal per day), while the CG was provided with a control diet (including average total carotenoids contents: 13.6 mg/2000 kcal per day). The experimental diet amount was provided in proportion to the design content according to the calories closest to the estimated energy requirements of each participant. In compliance with the Helsinki Declaration guidelines, the level of carotenoids in all diets was designed not to exceed the upper limit of vitamin A intake in consideration of the conversion of carotenoids to vitamin A, and the lowest level was above the estimated average requirement of Dietary Reference Intakes for Koreans (KDRI) [34].

After the 6-week diet intervention period, the participants were instructed to maintain their typical lifestyle habits and diet to detect changes during the 4-week tracking period. Restricting the carotenoid intake during the follow-up period could negatively affect health in research ethics; hence, rather than limiting the carotenoid intake during the follow-up period, they were instructed to return to a routine diet to ensure that it could return to the baseline level after a controlled diet. The details of the eligibility criteria and experimental diets are described in the Supplementary Materials (Supplemental Tables S1 and S2).

### 2.2. RRS-Based Measurement

**Figure 1:**
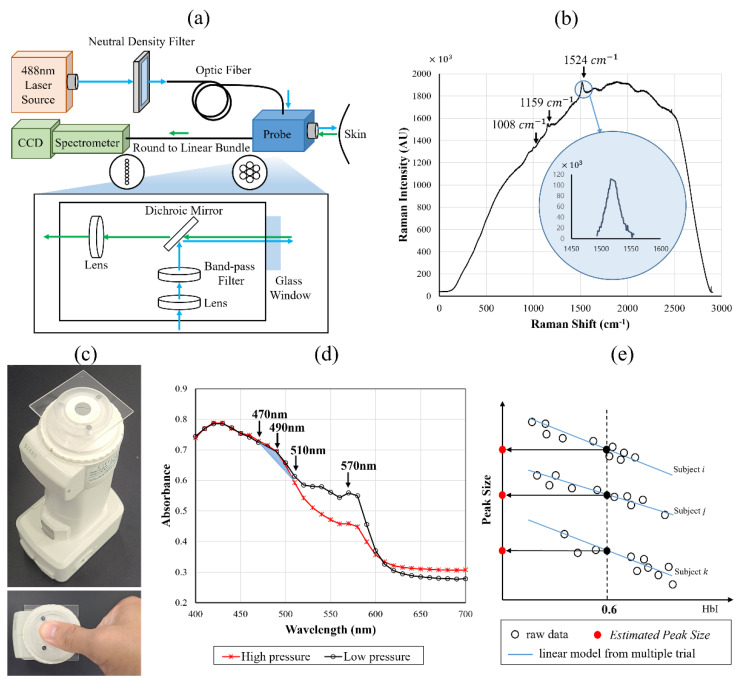
Devices and signals used in skin carotenoids level measurements are as follows: (**a**) Diagram of the RRS device used in this study; (**b**) a typical example of the skin Raman spectrum and an inset showing an enlarged area of the carotenoid Raman peak and post-processed Raman spectrum of the skin (in a blue circle). In the calculation of the skin carotenoid level, the most distinct peak at 1524 cm^−1^ was used. (**c**) Image of a commercial spectrophotometer (CM-700d), and (**d**) 490 nm peak observed from the in vivo spectrum. (**e**) Diagram for the calculation of the *Estimated Peak Size*. It is converted to ${Peak}_{RS}$ as expressed in Equation (2).

To evaluate SCL, a peak reflecting various types of carotenoids was used. As a reference, RRS has been widely used in previous studies because the size of the peak measured at the thumb was highly correlated with the SCL. To minimize the effect on other components of the skin, the right thumb, which has a low melanin concentration and a high carotenoid concentration due to its thick stratum corneum, was selected as the measurement site. The details of the peak size calculation using Raman spectra are described in a previous study [35]. The RRS device (Samsung Advanced Institute of Technology, Suwon, Republic of Korea) consisted of a 488 nm laser light source, a high-resolution spectrometer, and a charge-coupled device (CCD) camera (Figure 1a). The intensity of the laser was limited to approximately 2 × 10^3^ W/m^2^. The carotenoid-related peaks appear at the wavelengths of 1008 cm^−1^, 1159 cm^−1^, and 1524 cm^−1^. In the calculation of skin carotenoid levels, the area under the curve (AUC) of the Raman peak at 1524 cm^−1^ was calculated after the elimination of the background fluorescence (Figure 1b). The area was then normalized with the exposure time, accumulation number, and intensity of the laser. The peak size, in arbitrary units (AU), was designated ${Peak}_{RRS}$, and the value mostly ranged from 50 to 300, according to a previous study [35].

At each visit, the participants were instructed to conduct three trials by placing their right thumb in contact with the device’s glass window for measurement. For each trial, measurement with an exposure time of 400 ms was accumulated for 50 times. The average of the measured values was used for the analyses.

### 2.3. RS-Based Measurement

For the RS-based measurement, a commercial spectrophotometer (CM−700d, Konica Minolta, Inc., Tokyo, Japan) composed of an integrating sphere was used (Figure 1c). The commercial equipment used in this study is convenient in terms of its ease of operation, measurement time, and price compared to RRS. The device requires zero and white calibration with a provided white standard. Frequent calibration is not required, and monthly repeats are sufficient in a fixed environment. CM–SA Skin Analysis Software ver. 1.40.0001 (Konica Minolta, Inc., Tokyo, Japan) was used, and not only the raw reflectance of human skin but also the hemoglobin index (HbI), melanin index, and oxygen saturation index were provided [36,37]. Only raw reflectance data were used to evaluate the model in this study. This device, based on the integrating sphere structure, measured the reflection spectrum ranges from 400 nm to 700 nm (half bandwidth was approximately 10 nm). The specular component was included, and a glass plate of 1 mm thickness was fixed on the measurement site to prevent the convergence of the blood of the thumb. An aperture with a diameter of 8 mm was used in the MAV mode.

At each visit, participants were instructed to measure 10 trials that consisted of three repetitions of strong pressure, three repetitions of weak pressure, and four repetitions of arbitrary pressure using their right thumb. For each trial, fewer than 5 s were taken from the measurement click to the data acquisition. Because the pressure between the thumb and glass on the measurement site was negatively correlated with the blood volume, high pressure enables blood to diverge from the measurement site to minimize the noise due to the hemoglobin. However, the quantitative control of pressure is not useful because the relationship between physical pressure and the blood clearance may vary among individuals. Accordingly, HbI was monitored instead of pressure to distinguish between trials of strong and weak pressure. The vicinity of minimum and maximum HbI values indicate the conditions of strong and weak pressure, respectively. The maximum and minimum HbI values measured at each participant’s finger are clear. This study’s protocol was specifically designed to obtain data across a wide range of hemoglobin levels. On behalf of the HbI provided in the software, the absorbance at 570 nm (=${Abs}_{570}$) was used.

The wavelength range of the carotenoid spectrum is 400–510 nm, but in previous studies [20,25], the main peaks were selected within the 450–510 nm range to calculate only carotenoid concentrations while avoiding interference by other factors. To estimate the SCL using the RS method in this study, absorbance values for three wavelengths, 470 nm, 490 nm, and 510 nm, were used to calculate the *Peak Size*, as expressed in Equation (1). Because the RS spectrum measured on the thumb clearly shows the carotenoid-related peak near 490 nm (Figure 1d). ${Peak}_{RS}$ was calculated using a linear regression formula of RS and RRS derived from the data from the screening week, as expressed in Equations (1) and (2).

(1) $$Peak\;Size={Abs}_{490}-({Abs}_{470}+{Abs}_{510})/2$$

(2) $${Peak}_{RS}(\mathrm{AU})=Estimated\;Peak\;Size\times 8387.2+83.495$$

The peak size of Equation (1) is negatively correlated with HbI (Figure 1e), and the linear model was derived using the least-squares method. The linear model explains the relationship between HbI and the peak size. The *Estimated Peak Size (EPS)* is the result of the linear model at HbI = 0.6 and was defined as ${Peak}_{RS}$, which implies a peak size with a fixed hemoglobin effect. The *EPS* is converted to ${Peak}_{RS}$, as expressed in Equation (2).

### 2.4. Statistical Analysis and Evaluation

Correlation coefficients and *p* values were analyzed through Pearson correlation analyses for parametric data. Differences between the HG and the CG were assessed using *t*-tests for continuous variables and Chi-square tests or Fisher’s tests for categorical variables. For comparison of the two regression models, regression analysis with dummy variables was used, as expressed in Equation (3). If the intercept and slope have no difference, $a_{2}$ and $\beta_{2}$ are close to 0, and this would be not significant.

(3) $$Y_{t}=a_{1}+a_{2}D_{t}+\beta_{1}X_{t}+\beta_{2}\left(D_{t}X_{t}\right)+u_{t}$$

$$\mathrm{where}\;Y\;\mathrm{denotes}\;{Peak}_{RRS};\;X\;\mathrm{denotes}\;{Peak}_{RS};\;t\;\mathrm{represents}\;\mathrm{time};\;\mathrm{and}\;D\;=\;1\;\mathrm{for}\;\mathrm{observations}\;\mathrm{in}\;\mathrm{the}\;\mathrm{screening}\;\mathrm{week}\;\mathrm{and}\;D\;=\;0\;\mathrm{for}\;\mathrm{observations}\;\mathrm{at}\;\mathrm{baseline}$$

To confirm the change in SCL, a two-tailed paired *t*-test was applied. To compare the degree of change at each point of a timeline, the data used for the analysis included only the data of the participants who attended all the timelines. During the period of high-carotenoid diet intervention, the SCL was compared with that measured at week 1 (the week the intervention started). Similarly, during the tracking period, the SCL was compared with that measured at week 7 (the end of the diet intervention). All calculations were performed using MATLAB R2021b (Mathworks Inc., Natick, MA, USA).

### 2.5. Visualization Using a Difference Vector

To visualize the change in SCL for respective participants, difference vectors were calculated. The use of difference vectors in this study is described in Table 1. A vector consisting of two components, $\Delta {Peak}_{RRS}$ and $\Delta {Peak}_{RS}$, was identified and designated the difference vector. Both variables, ${Peak}_{RRS}$ and ${Peak}_{RS}$, should exhibit the same trend during the intervention and tracking periods, and the vector should be aligned parallel to the identity function. If both the variables increase, the vector points toward the top right, and in the opposite case, the vector points toward the bottom left. If the signs of $\Delta {Peak}_{RRS}$ and $\Delta {Peak}_{RS}$ differ, the angle with the identity function approaches π/2 rad. The size of the vector is scaled by a factor of 0.4 for visibility. If the angle value is close to 0 or π rad, the changes in size and direction for the two variables are interpreted to be the same. In the analysis using difference vectors, the participants who participated in all the measurements are included (listed in Supplemental Table S3).

### 2.6. Hit Rate

The proportion of participants whose ${Peak}_{RS}$ and ${Peak}_{RRS}$ changed as intended was calculated and defined as the hit rate. The use of the hit rate in the current study is described in Table 1. Even if a significant difference in SCL is confirmed between the two points of the timeline, it does not indicate that most participants commonly experienced a change in skin carotenoids. The significance of the *t*-test only guarantees the effectiveness of the diet intervention for each group. However, the hit rate enables the observation of the proportion of participants who have changed in the intended direction among all participants.

The chance level of the index was 0.5, which implies that the change was random. The probability of the hit rate was calculated to confirm significance, as shown in Equations (4)–(6). The variable n denotes the total number of participants, and k indicates the number of participants whose SCL changed as intended. The hit rate is calculated as k/n. Further, p is the probability that the SCL of the participant changes randomly in the intended direction. $f\left(k\right)$ of Equation (4) is the probability density function, and $F\left(k\right)$ in Equation (5) is the cumulative distribution function. $Pr\left(k\right)$ of Equation (6) is the probability that the SCL of k participants changed as intended. As k approaches the total number of participants n, $Pr\left(k\right)$ decreases. In the analysis using hit rates, all the participants with measurements are included (listed in Supplemental Table S3).

(4) $$f\left(k\right)=\left(\begin{array}{c} n\\ k \end{array}\right)p^{k}{\left(1-p\right)}^{n-k},\;\left(\begin{array}{c} n\\ k \end{array}\right)=\frac{n!}{k!\left(n-k\right)!}$$

(5) $$F\left(k\right)=\sum_{i=0}^{k}\left(\begin{array}{c} n\\ i \end{array}\right)p^{i}{\left(1-p\right)}^{n-i}$$

(6) $$Pr\left(k\right)=1-F(k-1)$$

### 2.7. Subgroup Classification

To compare the kinetics of the different initial SCL, we divided the participants into four groups on the basis of the SCL obtained using RRS in the screening week. The SCL obtained from the screening test ranged from approximately 50 AU to 250 AU, and the subgroups were divided into 50 AU intervals (Group 1 (G1): Over 200 AU, Group 2 (G2): 200–150 AU, Group 3 (G3): 150–100 AU, Group 4 (G4): Below 100 AU). In the HG, 5, 8, 14, and 8 participants belonged to groups G1, G2, G3, and G4, respectively. In the CG, 2, 10, 12, and 8 participants belonged to groups G1, G2, G3, and G4, respectively.

### 2.8. Sample Size

The effect size was considered in previous studies with regard to the effect of the carotenoid intake on skin and blood carotenoid levels [38]. The statistical parameters were applied as eleven repetition measurements with a correlation coefficient of 0.5, an effect size (f) of 31%, an alpha error (α) of 5%, and a power (1 − β) of 80%. G*Power software 3.1.9.7 [39,40] was used for the calculations; the minimum number of study participants was 48 (24 in the HG and 24 in the CG). Assuming that the general dropout rate of dietary intervention studies was 40%, a sample size of 80 participants (40 in the HG and 40 in the CG) was calculated.

## 3. Results

### 3.1. Participants’ Characteristics

Out of the total 152 individuals who enrolled in the study between December 2020 and February 2021, eight participants did not attend the screening test, 59 did not meet the inclusion criteria, and five declined to participate. The 80 eligible participants provided written and informed consent. All participants were randomly allocated to the high-carotenoid diet group (HG, n = 40) and control-carotenoid diet group (CG, n = 40) for the 6-week whole-diet intervention and 4-week tracking periods, from February to May 2021. Table 2 shows the characteristics of the participants at baseline. Finally, 72 participants completed the 10 weeks of research. This intervention study did not cause any significant harm to or unintended effects on the participants.

**Figure A2:**
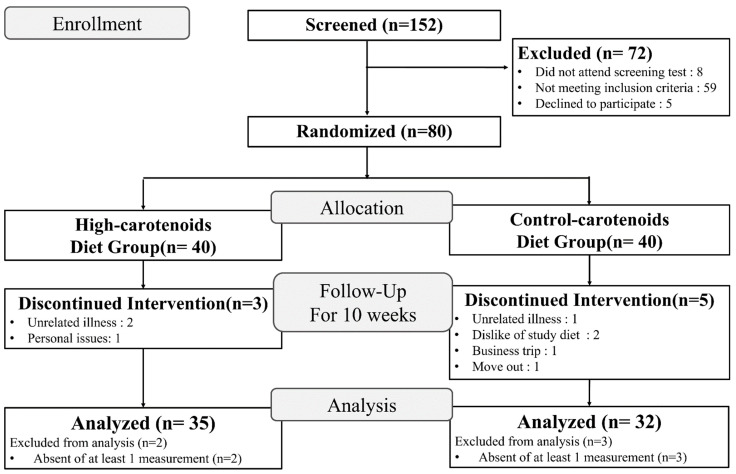
Flow diagram for the recruitment, randomization, and retention of study participants.

In the analysis, which was based on the average SCL or correlations between the two methods, RS and RRS, we included 67 participants who attended every week. Of these, 32 participants were part of the CG, while the remainder belonged to the HG. The analysis was conducted without any change in the group initially assigned. In the analysis using difference vectors or hit rates, all participants of each week were included (Supplemental Table S3). Details of the participant flow diagram are illustrated in Figure A2.

### 3.2. Correlation between RRS and RS

**Figure 2:**
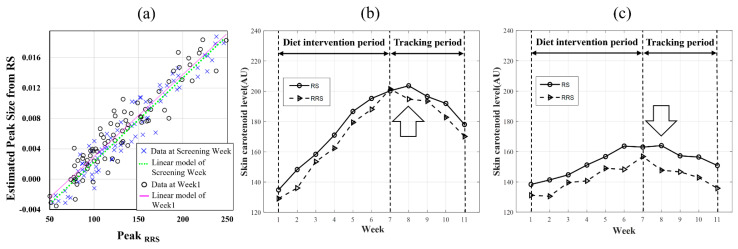
Changes in SCL at different timelines; (**a**) Comparison of SCL with the same participants (n = 80) measured at the screening week and week 1 (baseline). The *Estimated Peak Size* was measured using RS and calculated as represented in Figure 1. The slope of time interval measurements has no difference (*p*-value = 1.00). The average SCL of (**b**) the high–carotenoid diet group and (**c**) the control–carotenoid diet group, measured during the entire study. Observation was conducted for 10 weeks (6 weeks of the diet intervention and 4 weeks of the tracking). The arrows in Figure 2b,c indicate the time when the difference between the two methods occurs. AU, Arbitrary unit; SCL, Skin carotenoid level.

To verify the correlation of the SCL values obtained using RRS and RS and the agreement of the suggested model for RS, two datasets corresponding to RRS and RS obtained before the intervention were compared. The *EPS* was measured using RS and calculated as expressed in Equation (1). According to the data measured during the screening week, a positive correlation between the ${Peak}_{RRS}$ and *EPS* was observed (n = 80, r = 0.964, 95% confidence interval (CI) = 0.944–0.977, *p* < 0.001). Even in the data with time differences in five-week intervals, the correlation coefficient was observed to be similar to the value measured at week 1 (n = 80, r = 0.944, 95% CI = 0.914–0.964, *p* < 0.001). The data measured at the visits for the screening week and week 1 are presented in Figure 2a. The linear regression lines of the two timelines had no differences, as confirmed by the *t*-test (*p* > 0.99) and regression analysis with dummy variables (*p* = 0.16 of $a_{2}$ and *p* = 0.74 of $\beta_{2}$). The average SCL changes in ${Peak}_{RRS}$ and ${Peak}_{RS}$ were observed in a similar pattern for 10 weeks. However, for the RS approach, ${Peak}_{RS}$ took over three weeks to decrease significantly (*p* < 0.001), while ${Peak}_{RRS}$ took only one week (*p* = 0.004). Although the dietary intervention was terminated at week 7, elevated ${Peak}_{RS}$ scores were observed by week 8 in both the CG and HG (Figure 2b,c). Therefore, the correlation coefficient between ${Peak}_{RRS}$ and ${Peak}_{RS}$ was the lowest in week 7 (Table 3).

Table 3 shows the strong correlation coefficients between ${Peak}_{RRS}$ and ${Peak}_{RS}$ of 67 participants (n = 67 × 11 visits, r = 0.930, 95% CI = 0.919–0.939, *p* < 0.001) and in the HG and the CG (n = 35 × 11 visits, r = 0.919, 95% CI = 0.901–0.933 and n = 32 × 11 visits, r = 0.934, 95% CI = 0.919–0.946, all *p* < 0.001) for the entire study period. In each week, positively strong correlations (r = 0.916–0.935) were also confirmed in all participants, except for week 7 (r = 0.885), the end date of the diet intervention. Similarly, the correlations were high in each HG and CG (r = 0.857–0.931 and r = 0.928–0.953, respectively), except for week 7 (r = 0.798 and r = 0.898, respectively). Overall, a positive correlation between ${Peak}_{RRS}$ and ${Peak}_{RS}$ was evident (*p* < 0.0001).

In ${Peak}_{RRS}$, the average of standard deviations (SD) is 8.38, and the average of coefficient of variations (CV) is 5.68% during all periods (Supplemental Table S4). The SD and CV of the measured value were small enough compared to the changes in the trend, and the changes observed in the experiment were sufficient to overcome noise.

### 3.3. Change Rate between RRS and RS

The strength of the change was calculated as the slope from the beginning to the last point. During the intervention period in the HG, the ${Peak}_{RRS}$ increased by 12.4 AU/week (r = 0.996), while the ${Peak}_{RS}$ increased by 11.4 AU/week (r = 0.993) (Figure 2b). During the tracking period in the HG, the ${Peak}_{RRS}$ of the HG decreased by 7.5 AU/week (r = 0.962), while the ${Peak}_{RS}$ of the HG decreased by 5.6 AU/week (r = 0.891). During the intervention period in the CG, the ${Peak}_{RRS}$ increased by only 4.3 AU/week (r = 0.970), while the ${Peak}_{RS}$ increased by only 4.7 AU/week (r = 0.984) (Figure 2c). During the tacking period in the CG, the ${Peak}_{RRS}$ decreased by 4.7 AU/week (r = 0.968), while the ${Peak}_{RS}$ decreased by 3.2 AU/week (r = 0.941). Although the increase in the ${Peak}_{RRS}$ was 2.85 times greater in the HG, a significant increase was also observed in the CG. Similarly, the ${Peak}_{RS}$ also increased by 2.44 times more in the HG compared to the CG. In the tracking period, the decreases in the ${Peak}_{RRS}$ and ${Peak}_{RS}$ were 1.60 and 1.76 times greater, respectively, in the HG than in the CG.

The results of the paired *t*-test showed that both the ${Peak}_{RRS}$ and ${Peak}_{RS}$ of the HG increased significantly (*p* < 0.0001) after only one week of the diet intervention (Figure 2b). In the CG, two weeks were taken for a significant (*p* = 0.001 of ${Peak}_{RRS}$ and *p* < 0.001 of ${Peak}_{RS}$) increase compared to the baseline (Figure 2c). However, during the tracking period, significant changes were observed later in the ${Peak}_{RS}$ compared to the ${Peak}_{RRS}$. During the tracking period (Figure 2b,c), the ${Peak}_{RRS}$ decreased significantly one week after stopping the diet intervention in both the HG (*p* = 0.004) and the CG (*p* < 0.0001). During the same period (Figure 2b,c), the ${Peak}_{RS}$ decreased significantly three weeks after stopping the diet intervention in the HG (*p* = 0.002) and four weeks after in the CG (*p* < 0.001).

**Figure 3:**
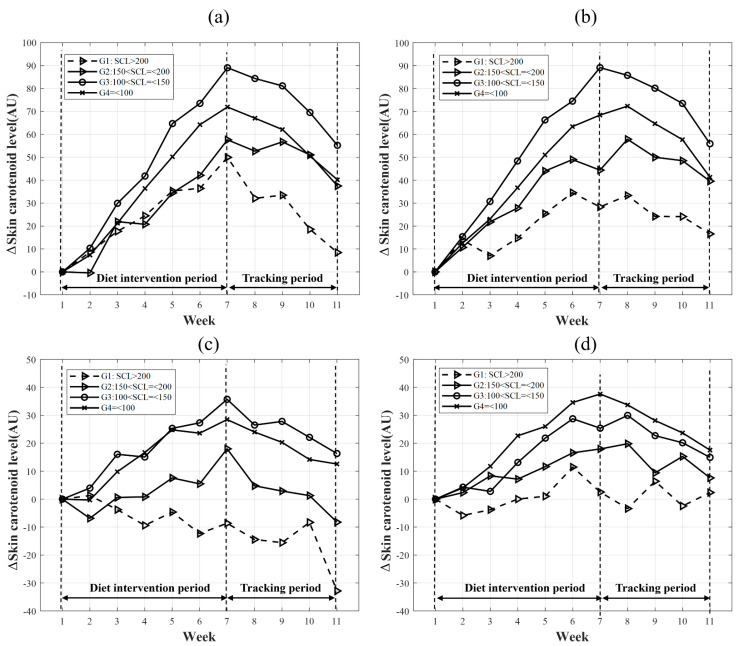
Average $\Delta {Peak}_{RRS}$ and ${\Delta Peak}_{RS}$ of subgroups G1–4 measured during the study from week 1. (**a**) $\Delta {Peak}_{RRS}$ of the HG. (**b**) ${\Delta Peak}_{RS}$ of the HG. (**c**) ${\Delta Peak}_{RRS}$ of the CG. (**d**) ${\Delta Peak}_{RS}$ of the CG. AU, Arbitrary unit; CG, Control-carotenoid diet group; HG, High-carotenoid diet group.

The changes in subgroups G1 to G4 divided by the criteria before the start of the study were compared. The SCL of the low-initial-level groups (subgroups G3 and G4) increased relatively faster than that of the high-initial-level groups (subgroups G1 and G2). During the intervention period, the ${Peak}_{RRS}$ of the HG increased by 50.0 AU, 57.6 AU, 89.1 AU, and 72.0 AU for groups G1, G2, G3, and G4, respectively (Figure 3a). The ${Peak}_{RS}$ of the HG increased by 28.3 AU, 44.4 AU, 89.1 AU, and 68.4 AU for groups G1, G2, G3, and G4, respectively, during the same period (Figure 3b). In the CG, the ${Peak}_{RRS}$ of group G1 decreased by 8.6 AU, while those of groups G2, G3, and G4 increased by 18.0 AU, 35.7 AU, and 28.5 AU (Figure 3c), respectively. The ${Peak}_{RRS}$ of groups G1, G2, G3, and G4 increased by 2.5 AU, 18.0 AU, 24.7 AU, and 37.6 AU, respectively (Figure 3d).

During the four weeks of tracking in the HG, ${Peak}_{RRS}$ decreased by 41.5 AU, 20.1 AU, 33.8 AU, and 31.8 AU for groups G1, G2, G3, and G4, respectively (Figure 3a). ${Peak}_{RS}$ decreased by 11.8 AU, 4.8 AU, 33.2 AU, and 27.1 AU for groups G1, G2, G3, and G4, respectively (Figure 3b). In the CG, the ${Peak}_{RRS}$ of groups G1, G2, G3, and G4 decreased by 24.2 AU, 26.2 AU, 19.4 AU, and 15.9 AU, respectively (Figure 3c). The ${Peak}_{RS}$ of groups G1, G2, G3, and G4 decreased by 0.2 AU, 10.4 AU, 10.4 AU, and 20.1 AU, respectively (Figure 3d).

### 3.4. Vector Field between RRS and RS

**Figure 4:**
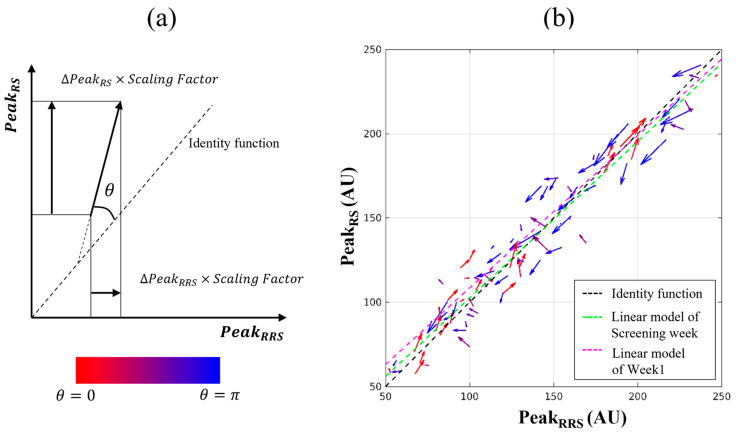
Vector field; (**a**) Concept of the difference vector consisted of two components, $\Delta {Peak}_{RRS}$ and $\Delta {Peak}_{RS}$. As the difference angle (=arrow) approaches 0 rad, the vector is expressed stronger in red. As the difference angle approaches π rad, the vector is expressed stronger in blue. A scaling factor of 0.4 was applied for a proper visualization. (**b**) Difference vectors of data measured at the screening week and week 1 exhibit various trends before intervention. In the y-axis, the *Estimated Peak Size* was converted to a Raman scale according to Equation (2) as ${Peak}_{RS}$. It shows effectively in visualizing changes in time intervals at individual levels. AU, Arbitrary unit.

A vector field comprises the difference vectors defined in Figure 4a. The details of the difference vector were described in Section 2.5. The angle with the identity function was calculated to quantify the agreement between the $\Delta {Peak}_{RRS}$ and $\Delta {Peak}_{RS}$. Then, the arrow of the vector was colored to express its direction. As the difference angle approaches 0 rad, the vector is expressed stronger in red. As the difference angle approaches π rad, the vector is expressed stronger in blue. A scaling factor of 0.4 was applied for a proper visualization. In the y-axis, ${Peak}_{RS}$ was converted to the RRS scale according to Equation (2). Because the data in Figure 4b were measured before the intervention, various trends of difference vectors were observed. While the conventional scatter plot (Figure 2a) focused on the correlation and distribution of data, the vector field has shown the individual SCL changes of each participant in one chart.

**Figure 5:**
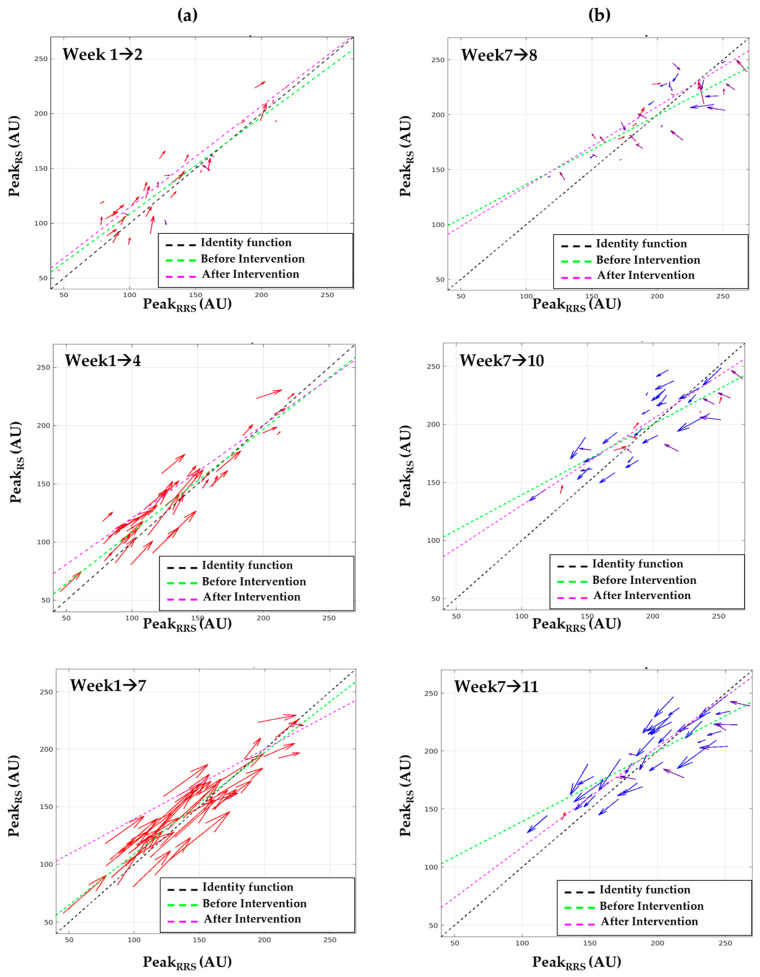
(**a**) As a vector field during carotenoid diet intervention period, the starting point of the vector was [${Peak}_{RRS}$, ${Peak}_{RS}$] at week 1. (**b**) As a vector field during tracking period in the high-carotenoid diet group, the starting point of the vector was [${Peak}_{RRS}$, ${Peak}_{RS}$] at week 7. As the difference angle approaches 0 rad, the vector (=arrow) is expressed stronger in red. As the difference angle approaches π rad, the vector is expressed stronger in blue. A scaling factor of 0.4 was applied for a proper visualization. AU, Arbitrary unit.

By employing a differences vector, Figure 5 illustrates the behavior of individual participants’ SCL through a comparison of the two methods, RS and RRS. In Figure 5a, vector fields for the 6-week diet intervention (weeks 1 to 7) in the HG are plotted. The difference vectors consisted of two components, $\Delta {Peak}_{RRS}$ and $\Delta {Peak}_{RS}$, and the starting point of the vector was [${Peak}_{RRS}$, ${Peak}_{RS}$] at week 1. The respective vectors were gradually aligned to the upper right, and the length of the vectors increased. This shows that most participants experienced an increase in both ${Peak}_{RRS}$ and ${Peak}_{RS}$ simultaneously. Particularly, ${Peak}_{RRS}$ and ${Peak}_{RS}$ increased simultaneously for all participants over time. In Figure 5b, the vector fields for the 4-week tracking period (weeks 7 to 11) were plotted, and the starting point of the vector was [${Peak}_{RRS}$, ${Peak}_{RS}$] at week 7. Although almost all participants experienced a decrease in ${Peak}_{RRS}$ and ${Peak}_{RS}$, the vectors of a few participants were not well aligned with the identity function. In week 8, the tendency for the vectors to decrease to the lower left was not observed. However, an overall downward trend was shown in week 10, although several exceptional participants remained.

### 3.5. Trend of Angles between RRS and RS

**Figure 6:**
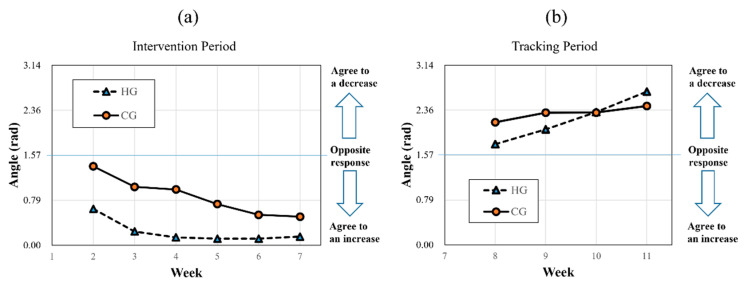
Average trend of the angle of the high-carotenoid diet group (HG) and control-carotenoid diet group (CG) in the period of (**a**) the diet intervention and (**b**) the tracking. The angle of (**a**) was calculated from the difference vector whose starting point is week 1, and the angle of (**b**) was calculated from the vector whose starting point is week 7. If the response of the RS and RRS methods is different, the angle approaches π/2 rad. When it approaches 0 rad, the RS and RRS methods agree in the direction of increasing, and when it approaches π rad, they agree in the direction of decreasing. AU, Arbitrary unit; CG, Control-carotenoid diet group; HG, High-carotenoid diet group.

Figure 6a shows the pattern approaching 0 rad over time when calculated from the difference vector with the starting point of week 1, indicating that the RS and RRS methods agreed in the direction of increasing (defined in Figure 4a). In week 2, the average angle was 0.635 rad, and it decreased to 0.138 rad in week 4. The angle reached an asymptote in three weeks during the intervention period of the SCL in the HG. The angle was maintained at less than 0.2 rad until the end of the intervention period (week 7). During the tracking period, the angle of (Figure 6b) was calculated from the vector whose starting point is week 7. The pattern of approaching 3.14 (=π) rad was confirmed by increasing the average angle to 2.68 in the HG, suggesting that the RS and RRS methods agreed in the direction of decreasing. In the case of CG, relatively slow changes were observed. During the intervention period, the angle reached 0.499 rad in week 7, and during the tracking period, it reached 2.42 rad in week 11. A saturation pattern was not observed for either the intervention or tracking period.

### 3.6. Hit Rates of RRS and RS

**Figure 7:**
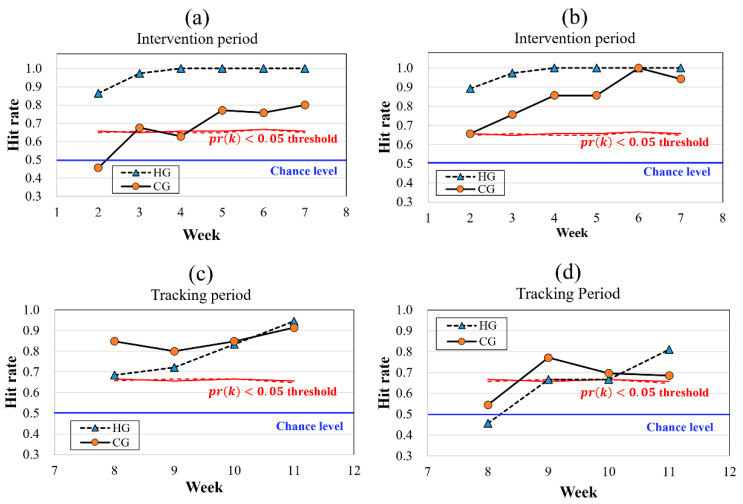
The hit rate, the proportion of participants that show the intended change in the carotenoid level, is presented. (**a**,**b**) show the hit rates of ${Peak}_{RRS}$ and ${Peak}_{RS}$ in the intervention period, respectively. (**c**,**d**) show the hit rates of ${Peak}_{RRS}$ and ${Peak}_{RS}$ in the tracking period, respectively. The chance level (*p* = 0.5) and the significance level ($Pr\left(k\right)<0.05$) are indicated with blue and red lines, respectively. Dashed and solid lines indicate the hit rates of the HG and CG, respectively. AU, Arbitrary unit; CG, Control-carotenoid diet group; HG, High-carotenoid diet group.

The hit rate, the proportion of participants that show the intended change in the carotenoid level, is presented in Figure 7. Hit rates were calculated and compared with the significance and chance levels (p=0.5). The minimum hit rates with $Pr\left(k\right)<0.05$ were calculated based on Equations (4)–(6) and are suggested as significant levels. In the HG, the carotenoid levels were increased by diet intervention in numerous individuals within one week. Both hit rates of ${Peak}_{RRS}$ in Figure 7a and ${Peak}_{RS}$ in Figure 7b exhibited similar tendencies. Increases in ${Peak}_{RRS}$ and ${Peak}_{RS}$ were measured in 0.865 and 0.891 of all participants in the first week (from weeks 1 to 2). At weeks 3 and 4, the hit rates of both ${Peak}_{RRS}$ and ${Peak}_{RS}$ approached 0.974 and 1.000, respectively. In the CG, the hit rate responded more immediately to ${Peak}_{RS}$ (Figure 7a,b) during the intervention period. Although the hit rate of ${Peak}_{RS}$ exceeded the significance level at week 3, that of ${Peak}_{RRS}$ did not overcome the significance level until week 5.

During the tracking period, the hit rates of ${Peak}_{RRS}$ exceeded the threshold from the first week (week 8) in both HG and CG (Figure 7c). The hit rates of ${Peak}_{RS}$ in the tracking period were not as high as those in other cases. This indicates that the carotenoid levels decreased in relatively fewer participants (Figure 7d).

## 4. Discussion

### 4.1. Principal Findings

This study entailed a whole-diet randomized controlled trial to evaluate the performance of a commercial RS-based spectrophotometer in measuring SCL as compared with RRS. Similar response patterns in the RRS and RS methods were identified using correlation analysis and the ratio of change during the intervention and tracking periods. Before the intervention, the correlation between ${Peak}_{RRS}$ and ${Peak}_{RS}$ was strongly confirmed in both the screening week and week 1 (n = 80, r = 0.964 and r = 0.944, respectively; both *p* < 0.001). Strong correlations were also observed over a 10-week study period in all participants, the HG, and the CG (r = 0.930, r = 0.919, and r = 0.934, respectively; all *p* < 0.001). This result is consistent with previous studies but shows a stronger correlation because the controlled diet was applied. The similarity of the change in ${Peak}_{RRS}$ and ${Peak}_{RS}$ was observed in each period. After 6 weeks of the intervention, the average increase ratios of the HG to the CG were 2.85 and 2.44 times for ${Peak}_{RRS}$ and ${Peak}_{RS}$, respectively. After 4 weeks of tracking, the average decrease ratios of the HG to the CG were 1.60 and 1.76 times for ${Peak}_{RRS}$ and ${Peak}_{RS}$, respectively. The results indicate that the dynamic behaviors based on RRS and RS are consistent.

When comparing the correlation by referring to RRS, the correlation with commercial devices (r = 0.92 to 0.96) in this study was higher than that of RS devices (r = 0.76 to 0.86) developed for SCL measurement [41]. The reason for the strong correlation with RRS is presumed to be the result of the proposed method enhancing the signal and reducing the noise with high selectivity for carotenoids while designing the study with a controlled diet. Moreover, existing studies using commercial spectrophotometers commonly performed poorly because they only used yellowness, which is the b* value of the Commission Internationale de l’Elcairage (CIE) color space, when measuring SCL [42,43]. However, to improve the accuracy of the RS method, we developed the SCL estimation model, which consists of three features (470 nm, 490 nm, and 510 nm) in the range of carotenoids and one feature (570 nm) in the range of blood. The effective cancelation of noise caused by the hemoglobin may contribute to revealing the apparent similarity between the two different modalities.

### 4.2. The Strengths of This Study

This study reported the characteristics of SCL changes and applied a novel analysis method to dynamic responses at the individual level. The current study revealed three distinctive tendencies for changes in the SCL. First, the change rate of the SCLs measured by both approaches depends on the initial level. In the case of participants with a low initial SCL, the difference between the amount of carotenoids intake before and after the intervention period begins is larger, which may result in a larger increase in ${Peak}_{RRS}$ and ${Peak}_{RS}$. This pattern was also observed in a previous study conducted with a small number of participants (n = 3) [21]. In that study, the SCL increased rapidly in the two subjects who started at a low SCL, but it increased slightly in the target with a high SCL during the intervention. Second, compared to the HG, the CG had less change in SCL, which can be interpreted as the number of carotenoids in the diet provided to the CG at a level similar to their usual diets. This suggests the possibility of quantifying the number of carotenoids in the daily intake based on the optically measured SCL. However, the initial SCL value may be affected by individual condition stress, gender, and obesity in addition to diet. Therefore, various factors should be controlled and closely observed for clear quantification [44]. Third, the rate of SCL decrease in the tracking period was slower than the rate of SCL increase in the intervention period. The carotenoid-rich diet resulted in a faster accumulation of carotenoids compared to their removal during the tracking period. This is similar to the dose-response phenomenon called “a clockwise hysteresis loop” or “tolerance” observed in the blood [45]. Carotenoids, which were delivered from the blood, accumulate quickly in the skin, but the rate of degradation in the skin was slower than in the blood [46].

Notably, the pattern of differences between RS and RRS in the initial tracking period suggests that ${Peak}_{RS}$ may reflect a longer-term dietary status than ${Peak}_{RRS}$, which responds relatively quickly to reduction. Existing studies [46,47,48,49,50] have shown that carotenoids move to the skin surface and that the concentration of carotenoids is higher on the skin surface than on the deep skin layer, which can lead to different results depending on the measured skin depth and area. The delayed reaction of RS at the time of reduction can be seen due to the RS characteristics of sampling being closer to the skin surface than RRS. The delay response in RS after the end of the intervention has also been found in another study [27].

Additionally, the new visualization tools and indices were proposed and confirmed for observing SCL changes from an individual rather than an average perspective. In previous studies, the effects of carotenoid-containing diets and supplement intake were confirmed by analyzing a trend of average values [17,20,21,27]. However, to the best of our knowledge, an observation of SCL change at an individual level has not yet been attempted. The hit rates of the significance level represented in Figure 7 indicate that both ${Peak}_{RRS}$ and ${Peak}_{RS}$ changed in the intended direction at a statistically significant ratio among all participants. For HG, the hit rates of ${Peak}_{RRS}$ and ${Peak}_{RS}$ show almost the same behavior. The condition of $Pr\left(k\right)<0.05$ was satisfied in one week, and hit rates gradually increase and reach 1.00 in three weeks (Figure 7a,b). This implies that a significant number of participants responded to a high-carotenoid diet in one week, and all participants exhibited intended SCL changes in three weeks. During the tracking period, the hit rate of RRS exceeded 0.90 in four weeks (Figure 7c), and it is not as rapid as the response in the intervention period. All these analyses indicate that SCL responded strongly in an intended manner to the intervention period in which the diet was controlled rather than to the tracking period in which the diet was relatively free.

Furthermore, the use of the vector fields was newly proposed to visualize the behavior of ${Peak}_{RRS}$ and ${Peak}_{RS}$ simultaneously for each participant (Figure 4a). It is an intuitive tool for observing agreement between the RS and RRS methods in response to intervention with one plot. Furthermore, it is an effective tool because it enables the visualization of not only the size of ${Peak}_{RRS}$ and ${Peak}_{RS}$ but also their agreement. If ${Peak}_{RRS}$ and ${Peak}_{RS}$ exhibit coincident behavior, the vector is arranged parallel to the identity function, which indicates the equivalence of ${Peak}_{RRS}$ and ${Peak}_{RS}$. The vectors of almost all participants are arranged parallel to the identity function, and this pattern is particularly rapid in the HG during the intervention period (Figure 5). This is quantitatively supported by the angle between the vector and the identity function, as shown in Figure 6. Therefore, these vectors, angles, and hit rates analysis tools can be an effective way to intuitively and quantitatively identify changes between two methods rather than depending on statistical analysis.

### 4.3. Limitations

A limitation of this study is that only participants with skin tones of an intermediate color were included. Therefore, an improved model for a general skin tone should be investigated in future studies. To cover the full range of skin tones such as Fitzpatrick skin types I to VI, the model should consider the effects of melanin, and the features with additional wavelengths should be designed. To resolve this issue, using RRS as a reference should be carefully considered because the approach is affected by chromophores [13]. The ideal solution is to acquire biopsies from skin tissue and measure the carotenoid content. The carotenoid content directly measured via HPLC of the biopsy can be used to estimate the parameters of the improved RS-based model.

To quantify the usual carotenoid intake, it is necessary to further examine whether the relationship between the equilibrium SCL and usual intake exists. However, both the ${Peak}_{RRS}$ and ${Peak}_{RS}$ of the HG increased almost linearly during the intervention period, and the saturation point was not observed in the current study. Casperson et al. [51] also conducted experiments on an additional carotenoid intake at three different levels (13.1 mg/day, 23.9 mg/day, and 31.0 mg/day) for eight weeks; however, their design conditions were also not sufficient to observe the saturation point of skin carotenoid change. Therefore, it is necessary to investigate the saturation point through a longer intervention period or by examining high-dose responses to natural food.

## 5. Conclusions

Compared to RRS, RS-based commercial equipment is useful because commercial RS has faster measurement times without calibration every time, is convenient to measure at reasonable prices, and is simple to implement. Therefore, a commercial device applying the proposed method is expected to be used as an alternative to conventional RRS in future applications for SCL measurement. Consequently, the results of this study enable more convenient V & F intake monitoring and will contribute to an improvement of dietary management for personal health.

## 6. Patents

SAMSUNG ELECTRONICS Co., Ltd. (Suwon, Republic of Korea) has a patent (Patent No.: US11359965) for RS-based skin carotenoid level measurement.

## Figures and Tables

**Table 1:** Analysis methods for agreement between RS and RRS.

Comparison Factors	Previous Study	Additional Analysis in This Study
Cross-sectional value	Correlation coefficient	No additional analysis
Response to intervention	Change in average	Angle of difference vector
Visualization	Scatter plot (Cross-sectional)	Hit rate

**Table 2:** Baseline characteristics of the study participants ^a^.

		Total	HG	CG	*p* Value ^b^
		(n = 80)	(n = 40)	(n = 40)	
Skin carotenoid level (AU) by					
	Resonance Raman spectroscopy (RRS)	129.0 ± 47.3	127.9 ± 44.6	130.2 ± 50.4	0.83
	Reflection spectroscopy (RS) ^c^	134.8 ± 45.4	133.6 ± 42.6	136.0 ± 48.5	0.82
Demographic and physical characteristics					
	Age (years)	32.0 ± 7.4	32.0 ± 7.4	32.1 ± 7.5	0.93
	Male/Female (n)	42/38	21/19	21/19	>0.99
	BMI (kg/m^2^)	27.1 ± 3.2	27.0 ± 3.3	27.1 ± 3.3	0.85
	Waist circumference (cm)	91.2 ± 8.5	90.6 ± 8.5	91.8 ± 8.6	0.55

^a^ Values are means ± standard deviations for continuous variables. ^b^ Differences between HG and CG were assessed using *t*-tests for continuous variables and Chi-square tests of Fisher’s tests for categorical variables. ^c^ Application of SCL unit calculated by RRS. CG, Control–carotenoid diet group; HG, High–carotenoid diet group; BMI, Body mass index.

**Table 3:** Correlation coefficients (95% CI) between ${Peak}_{RRS}$ and ${Peak}_{RS}$ during the entire study period ^a^.

	All Participants	HG	CG
	(n = 67)	(n = 35)	(n = 32)
Week 1 ^b^ (n = 80)	0.944 (0.914–0.964)	0.942 (0.892–0.969)	0.946 (0.899–0.971)
Week 1	0.933 (0.893–0.958)	0.931 (0.866–0.965)	0.936 (0.871–0.968)
Week 2	0.935 (0.895–0.959)	0.926 (0.858–0.962)	0.942 (0.883–0.972)
Week 3	0.933 (0.893–0.958)	0.918 (0.842–0.958)	0.953 (0.905–0.977)
Week 4	0.931 (0.89–0.957)	0.914 (0.834–0.956)	0.941 (0.882–0.971)
Week 5	0.932 (0.891–0.958)	0.918 (0.842–0.958)	0.928 (0.857–0.965)
Week 6	0.921 (0.874–0.951)	0.857 (0.733–0.926)	0.949 (0.897–0.975)
Week 7	0.885 (0.819–0.928)	0.798 (0.633–0.894)	0.898 (0.801–0.95)
Week 8	0.926 (0.882–0.954)	0.868 (0.752–0.932)	0.939 (0.878–0.97)
Week 9	0.927 (0.884–0.955)	0.889 (0.79–0.943)	0.934 (0.868–0.968)
Week 10	0.925 (0.88–0.953)	0.867 (0.75–0.931)	0.944 (0.888–0.973)
Week 11	0.916 (0.867–0.948)	0.888 (0.788–0.942)	0.939 (0.877–0.97)
All periods (Week 1~11)	0.930 (0.919–0.939)	0.919 (0.901–0.933)	0.934 (0.919–0.946)

^a^ Correlation coefficients and *p* values were analyzed through Pearson correlation analyses. The data of 67 participants (n = 35 in the HG; n = 32 in the CG) with all measurements were used for the analysis of 1 to 11 weeks and all periods. ^b^ At week 1 (baseline), data from all participants (n = 80) and randomly allocated to the high-carotenoid diet group (HG, n = 40) and control-carotenoid diet group (CG, n = 40) were used for analysis. All data were observed at a *p* value < 0.001. CG, Control-carotenoid diet group; CI, Confidence Interval; HG, High-carotenoid diet group.

## Data Availability

The data that support the findings of this study are available from the first and corresponding authors upon reasonable request. Furthermore, all data are the property of SAMSUNG ELECTRONICS Co., Ltd., and permission is required to export it.

## Supplementary Materials

The following supporting information can be downloaded at: https://www.mdpi.com/article/10.3390/s23177654/s1, Table S1: Eligibility criteria for the in vivo study; Table S2: Contents of daily nutrients and carotenoids in the experiment diets; Table S3: Number of participants during the whole period. Table S4: The average of standard deviations (STD) and coefficient of variations (CV) in RRS measurements during all study periods.
