# Supplementary material for: Evaluation of a Commercial Device Based on Reflection Spectroscopy as an Alternative to Resonance Raman Spectroscopy in Measuring Skin Carotenoid Levels: Randomized Controlled Trial
Source: Sensors (Basel). 2023 Sep 4;23(17):7654. doi: 10.3390/s23177654 (PMC10490775; doi:10.3390/s23177654)
Supplement: Supplementary file 1 [file sensors-23-07654-s001.zip › sensors-2578045-supplementary.pdf]

Supplementary documents.

Table S1. Eligibility criteria for the in vivo study

Criteria	Description
Inclusion	Koreans over 18 years old; no limit<br>Those who meet all of the following conditions can apply:<br>① People with a body mass index ≥ 23 kg/m2 or people with a fasting blood glucose ≥ 100 mg/dL without an underlying disease<br>② People who do not take medications for the control of blood glucose, cholesterol, or blood pressure<br>③ Korean adults aged ≥ 18 years
Exclusion	Those who meet any of the following criteria will be excluded:<br>① People with underlying diseases; diagnosed with diabetes, dyslipidemia, or high blood pressure; taking medications or receiving treatment for the abovementioned diseases; receiving treatment for cardiovascular, cerebrovascular, kidney, liver, intestinal, or endocrine disorders; or having skin diseases or taking medications for skin disease treatment<br>② People who have taken dietary supplements or antibiotics or probiotics in the past 3 months<br>③ People who have unusual eating habits or people who are allergic to certain foods<br>④ People who have smoked in the past 1 year<br>⑤ Pregnant or lactating women

Table S2. Contents of daily nutrients and carotenoids in the experiment diets [1]

	High-carotenoid diet	Control-carotenoid diet		
	Designed[1]	Analytical[1]	Designed	Analytical
Energy and nutrients				
Energy (kcal)	1997.7	1720.94	1991.4	1739.0
Carbohydrates (g)	295.6	274.6	290.6	275.4
Carbohydrates (% of energy)	59.5	63.8	58.7	63.3
Protein (g)	75.3	62.9	75.4	72.2
Protein (% of energy)	15.1	14.6	15.2	16.6
Fat (g)	56.2	41.2	57.3	38.8
Fat (% of energy)	25.4	21.6	26.0	20.1
Cholesterol (mg)	252.6	173.1	255.0	175.4
Fiber (g)	29.5	25.3	28.2	35.2
Vitamin C (mg)	139.7	23.3	136.8	9.9
Vitamin E (mg)	27.1	24.0	26.2	23.9
Calcium (mg)	562.3	474.8	538.7	466.4
Potassium (mg)	3463.1	2513.7	3165.6	2320.5
Magnesium (mg)	123.9	292.7	126.3	277.8
Sodium (mg)	2158.8	2976.7	2114.9	2474.4
Carotenoids and vitamin A				
Total carotenoids (mg) [2]	25.7	21.0	9.2	13.6
α-carotene (mg)	5.61	1.4	0.66	0.43
β-carotene (mg)	12.2	11.5	4.5	5.2
Lycopene (mg)	3.04	1.00	0.60	0.74
β-cryptoxanthin (mg)	0.35	0.5	0.30	0.37

Lutein & Zeaxanthin (mg)	4.45	6.6	3.17	6.8
Retinol (mg)	0.13	0.07	0.13	0.08
Vitamin A (μg RAE) [3]	1396.6	1105.6	545.7	553.0

[1] Contents of energy and nutrients were estimated using the CAN-Pro 5.0. Contents of carotenoids and vitamin A were estimated using the vitamin A database for common Korean foods. Contents of nutrition and carotenoids were analyzed by high-performance liquid chromatography and standard protocols.

[2] Sum of α-/β-carotene, lycopene, a/β-cryptoxanthin, lutein, and zeaxanthin contents

[3] Vitamin A (μg RAE) = μg retinol + μg β-carotene/12 + μg α-carotene/24 + μg β-cryptoxanthin/24. RAE, retinol activity equivalent.

Table S3. Number of participants during the whole period

Week	1	2	3	4	5	6	7	8	9	10	11	Participated all weeks
CG	40	35	37	35	35	33	35	33	35	33	35	32
HG	40	37	38	37	37	36	37	35	36	36	37	35
Total	80	72	75	72	72	69	72	68	71	69	72	67

CG, Control-carotenoid diet group; HG, High-carotenoid diet group

Table S4. The average of standard deviations (STD) and coefficient of variations (CV) in RRS measurements during all study periods.

	Number of participants	Average of STD in each participant	Average of CV (%) in each participant
**All**	**-**	**8.38**	**5.68**
Week 1	80	6.86	5.87
Week 2	72	7.76	6.24
Week 3	75	7.71	5.61
Week 4	72	8.40	6.06
Week 5	72	7.73	4.98
Week 6	69	8.60	5.28
Week 7	72	9.84	5.56
Week 8	68	7.95	5.00
Week 9	71	7.60	4.88
Week 10	69	12.35	7.90
Week 11	72	7.36	5.10
